# Foxtail Millet Improves Blood Glucose Metabolism in Diabetic Rats through PI3K/AKT and NF-κB Signaling Pathways Mediated by Gut Microbiota

**DOI:** 10.3390/nu13061837

**Published:** 2021-05-27

**Authors:** Xin Ren, Linxuan Wang, Zenglong Chen, Dianzhi Hou, Yong Xue, Xianmin Diao, Qun Shen

**Affiliations:** 1 Beijing Advanced Innovation Center for Food Nutrition and Human Health, Beijing Engineering and Technology Research Center of Food Additives, Beijing Technology and Business University, Beijing 100048, China; renxin@btbu.edu.cn (X.R.); wlinxuan0729@163.com (L.W.); 2 Key Laboratory of Plant Protein and Grain Processing, National Engineering and Technology Research Center for Fruits and Vegetables, College of Food Science and Nutritional Engineering, China Agricultural University, Beijing 100083, China; xiaozhihou90@126.com (D.H.); xueyong@cau.edu.cn (Y.X.); 3 State Key Laboratory of Integrated Management of Pest Insects and Rodents, Institute of Zoology, Chinese Academy of Sciences, Beijing 100101, China; chenzenglong@ioz.ac.cn; 4 Center for Crop Germplasm Resources, Institute of Crop Science, Chinese Academy of Agricultural Sciences, Beijing 100081, China; diaoxianmin@caas.cn

**Keywords:** foxtail millet, glucose metabolism, gut microbiota, PI3K/AKT signaling pathway, NF-κB signaling pathway

## Abstract

Foxtail millet (FM) is receiving ongoing increased attention due to its beneficial health effects, including the hypoglycemic effect. However, the underlying mechanisms of the hypoglycemic effect have been underexplored. In the present study, the hypoglycemic effect of FM supplementation was confirmed again in high-fat diet and streptozotocin-induced diabetic rats with significantly decreased fasting glucose (FG), glycated serum protein, and areas under the glucose tolerance test (*p* < 0.05). We employed 16S rRNA and liver RNA sequencing technologies to identify the target gut microbes and signaling pathways involved in the hypoglycemic effect of FM supplementation. The results showed that FM supplementation significantly increased the relative abundance of Lactobacillus and Ruminococcus_2, which were significantly negatively correlated with FG and 2-h glucose. FM supplementation significantly reversed the trends of gene expression in diabetic rats. Specifically, FM supplementation inhibited gluconeogenesis, stimulated glycolysis, and restored fatty acid synthesis through activation of the PI3K/AKT signaling pathway. FM also reduced inflammation through inhibition of the NF-κB signaling pathway. Spearman’s correlation analysis indicated a complicated set of interdependencies among the gut microbiota, signaling pathways, and metabolic parameters. Collectively, the above results suggest that the hypoglycemic effect of FM was at least partially mediated by the increased relative abundance of Lactobacillus, activation of the PI3K/AKT signaling pathway, and inhibition of the NF-κB signaling pathway.

## 1. Introduction

Diabetes and its associated disorders have reached an alarming level worldwide. In 2019, an estimated 463 million adults aged 20–79 years old worldwide had diabetes, and by 2045, 700 million adults will be living with diabetes [1]. Type 2 diabetes (T2D) accounts for the vast majority of diabetes. Recent decades have seen an exponential increase in the number of people suffering from T2D, despite the expanding number of anti-hyperglycemic medication options [2]. Fortunately, there is firm evidence that T2D can be prevented and effectively managed through the adoption of healthy lifestyles [1], including the increased consumption of whole grains [3].

Foxtail millet (*Setaria italica* L., FM) was arguably the first whole grain cultivated by humans [4]. Currently, it is the sixth-highest yielding grain in terms of worldwide production and is cultivated in 26 countries [5]. FM contains significant levels of protein, fiber, minerals, phenolic acids, and various phytochemicals. It has received ongoing increased attention, particularly due to its hypoglycemic, hypolipidemic, and antioxidant characteristics [4,6]. Many studies, including our previous work, have proven that FM has a lower glycemic index, which is conducive to the glycemic control of patients with abnormal blood glucose [7,8]. However, the mechanisms underlying the hypoglycemic effect of FM were still unclear.

Recent studies have provided a substantial body of evidence for the contribution of the gut microbiota to glucose metabolism [9,10]. It is generally believed that the occurrence and development of T2D is one result of gut microbial dysbiosis caused by an unbalanced diet [11]. Research suggested that changes in the diet could account for 57% of the variations in microbiota [12]. FM could indeed change the composition and relative abundance of gut microbiota. For instance, Li et al. found that FM supplementation decreased the population of Firmicutes and increased Actinobacteria in rats fed a high-fat diet [13].

However, the exact contribution of the gut microbiota to the hypoglycemic effect of foxtail millet is still not clear due to the complexity and diversity of gut microbes. A number of mechanisms by which gut microbiota may influence glucose metabolism have been investigated, such as the insulin signaling pathway and low-grade inflammation [14]. Specifically, the insulin-mediated PI3K/AKT signaling pathway and inflammatory factor-mediated NF-κB signaling pathway are two key processes in T2D. Many studies have proved that glucose metabolism, including glycolysis and gluconeogenesis, is mainly regulated by the PI3K/AKT signaling pathway [15]. The hypoglycemic function of bioactive substances and hypoglycemic drugs cannot be achieved without the participation of this pathway [16,17]. For example, the PI3K/AKT signaling pathway can inhibit the key enzymes of gluconeogenesis and thus play an important role in maintaining the homeostasis of glucose metabolism [18]. T2D is associated with low-grade inflammation [19], and the NF-κB signaling pathway is the primary method of the inflammatory response [20].

Thus, in the present study, 4 weeks of FM intervention was conducted in high-fat diet/streptozotocin (HFD/STZ)-induced diabetic rats. In addition to glycemic metabolism indicators, 16S rRNA and RNA sequencing technologies were employed to investigate the differences between the gut microbiota and liver transcriptome in diabetic rats. We evaluated the key gut microbe and liver signaling pathways affected by FM supplementation. The target genes and target biological processes of foxtail millet in improving glucose metabolism were identified. Finally, the mechanisms underlying the hypoglycemic effect of FM were partially clarified.

## 2. Materials and Methods

### 2.1. Animals and Diet

FM was provided by Shanxi Dongfangliang Life Sciences Co., Ltd. To maintain consistency with our previous clinical trial [8], the FM was processed into steamed bread according to the previous introduction [7]. After being freeze-dried and crushed, the powder of FM steamed bread was added to rat feed at a rate of 20%, which was similar to the intervention amount of subjects in a clinical trial [8]. The specific formulae for the different diets are listed in Supplementary Materials Table S1. The energy ratio between the 20% FM diet and the high-fat diet was equal.

Male SD rats (6 weeks old) were obtained from Vital River Laboratories Co., Ltd. (Beijing, China, SCXK (J) 2016-0006). They were kept in a climate-controlled room (22 ± 2 °C, 55% ± 5% relative humidity, and a 12 h light/dark cycle) with free access to food and water. All animal procedures were conducted according to the guidelines of the Laboratory Animal Ethics Association of China Agricultural University.

After one week of acclimatization, eight rats were randomly grouped into the normal control (NC) group and fed with the D12450J control diet (Research Diets, New Brunswick, NJ, USA). All the other rats were induced to diabetics by 4 weeks of a high-fat diet (D12492) and 35 mg/kg of STZ injection. Then, the diabetic rats were randomly divided into the diabetic control (DC) group and FM group with eight rats each for four weeks of intervention. The body weight and food intake of rats were recorded weekly. At the end of the intervention, rats were euthanized by decapitation under isoflurane anesthesia. Blood samples were collected and centrifuged at 3000 r/min for 10 min. The serum, liver tissues, and feces were collected and stored at −80 °C until analysis. 

### 2.2. Biochemical Analysis

The serum concentrations of the fasting glucose (FG), total triglycerides (TG), total cholesterol (TC), and high-density lipoprotein cholesterol (HDL-C) were measured using a COBAS INTEGRA 800 auto-analyzer (Roche, Basel, Switzerland) per the manufacturer’s protocols. The fasting insulin (ml302840), glycated serum protein (GSP, ml037457), glucose kinase (GK, ml059525), glucose-6-phosphatase (G6P, ml196120), and phosphoenolpyruvate carboxy (PEPCK, ml059012) were determined using commercial kits (Shanghai Enzyme-linked Biotechnology Co., Ltd., Shanghai, China). Then, via homeostasis model assessment, the insulin resistance index (HOMA-IR) was calculated [21].

In addition, i.p. glucose tolerance tests (GTTs) were performed three days before the end of the experiment. In brief, after a 12 h fast, all rats were administered a 50% glucose solution (2.0 g/kg body weight), and blood samples from the tail veins at 0, 30, 60, 120, and 180 min were collected successively to measure the blood glucose concentration.

### 2.3. Gut Microbiota Analysis

The gut microbiota of rats were investigated after 4 weeks of intervention, according to the method described before [22]. In brief, the total bacterial DNA was extracted from fecal samples using MoBio Power Soil HTP-96 extraction kits (MoBio Laboratories, Carlsbad, CA, USA). The 338F (5′-ACTCCTACGGGAGGCAGCAG-3′) and 806R (5′-GGACTACHVGGGTWTCTAAT-3′) primer pair targeting the V3–V4 region of the 16S rRNA gene was chosen for PCR amplification. The resulting PCR products were purified using a QIAquick PCR Purification Kit (QIAGEN, Valencia, CA, USA). Finally, qualified DNA samples were sequenced and analyzed on an Illumina MiSeq platform (Illumina, Inc., San Diego, CA, USA).

### 2.4. RNA Sequencing (RNA-Seq) Analysis

The total RNA was extracted from liver tissue using TRIzol reagent (Invitrogen, Thermo Scientific, MA, USA) following the manufacturer’s instructions. The quantity and quality of RNA were determined using a NanoDrop 2000 ultramicro-spectrophotometer (ThermoFisher Scientific, Waltham, MA, USA). The RNA integrity was estimated using an RNA 6000 Nano Bioanalyzer 2100 Assay (Agilent, Palo Alto, CA, USA). Library construction and sequencing were performed with the Illumina HiSeq platform by Majorbio Biopharm Technology (Shanghai, China). 

Expression profiles were obtained using the free online platform of Majorbio Cloud Platform (www.majorbio.com) (accessed on 20 January 2021). In brief, the fold changes were estimated according to the fragments per kilobases per million reads (FPKM) in each sample, and differential expression analysis was performed with the DESeq2 package. We considered differentially expressed genes (DEGs) with *p* < 0.05 and |log2 FC| > 1. Finally, enrichment analysis was performed using the Kyoto Encyclopedia of Genes and Genomes (KEGG) database.

### 2.5. Real-Time PCR (RT-PCR) Analysis

The total RNA was extracted from liver tissues with TRIzol reagent (Invitrogen, Carlsbad, CA, USA) according to the manufacturer’s instructions. RNA was then reverse transcribed into cDNA using a RevertAid First cDNA Synthesis Kit (K1622, Thermo Scientific, CA, USA). RT-PCR was performed using SYBR Green dye for the relative quantification of DEG expression. The relative mRNA expression levels of the genes were calculated by the 2^−^^△△CT^ formula, and β-actin was used as the housekeeping gene. The primers are listed in Table S2.

### 2.6. Western Blot Analysis

Liver tissue was lysed in lysis buffer (100 mg:1 mL) supplemented with protease inhibitor. After determining the protein concentration, the lysates were separated on SDS-PAGE gels and transferred to 0.22 μm PVDF membranes. The membranes were blocked in PBS containing 1% Tween-20 and 5% milk for 1 h at 37 °C and incubated with primary antibodies (Beyotime Biotechnology, Shanghai, China) overnight at 4 °C. The specific primary antibodies used in the present study were AKT (60KD, AA326), p-AKT (Ser473, 60KD, AA329), NF-κB-p65 (65KD, AN365), p-NF-κB-p65 (Ser536, 65KD, AN371), p-IΚBα (Ser32, 36KD, AF1870) and p-IKKα/β (Ser176/180, 86KD/87KD, AI139). After 1 h of incubation at 37 °C with goat anti-rabbit secondary antibodies (SA00001-2, Proteintech Group Inc., Chicago, IL, USA), Western blot images were captured using a Tanon-3500 gel imaging system (Tanon, Shanghai, China).

### 2.7. Immunofluorescence Staining

Immunofluorescence staining was performed on 5 μm thick paraffin sections of isolated liver tissues fixed in paraformaldehyde. Sections were deparaffinized in xylene and rehydrated in graded alcohols. Endogenous peroxidase was blocked, followed by antigen retrieval. After this, the tissues were incubated with the primary antibodies (p-NF-κB-p65, AN371, Beyotime Biotechnology, Shanghai, China) overnight at 4 °C, and then incubated with FITC modified second antibody (A0562, Beyotime Biotechnology, Shanghai, China) for 1 h at 37 °C. The nuclei were counterstained with DAPI. The images were captured using confocal scanning laser microscopes. 

### 2.8. Statistical Analysis

Statistical analyses were conducted using SPSS software version 20.0 (IBM Corp., Armonk, NY, USA), and graphs were plotted using GraphPad Prism (GraphPad Software, San Diego, CA, USA). The data of biochemical, gut microbiota, RT-PCR, and Western blot were represented as the mean ± standard deviation (SD) and preanalyzed using Shapiro–Wilk test. The data of RNA-Seq were estimated according to FPKM. Differences between the two groups were compared using Student’s *t*-test. Differences between the three groups were compared using one-way ANOVA with Tukey’s multiple comparison post hoc test. All statistical tests were two-sided. Differences with *p* < 0.05 were considered statistically significant.

## 3. Results

### 3.1. FM Supplementation Improved the Blood Glucose Metabolism

**Figure 1:**
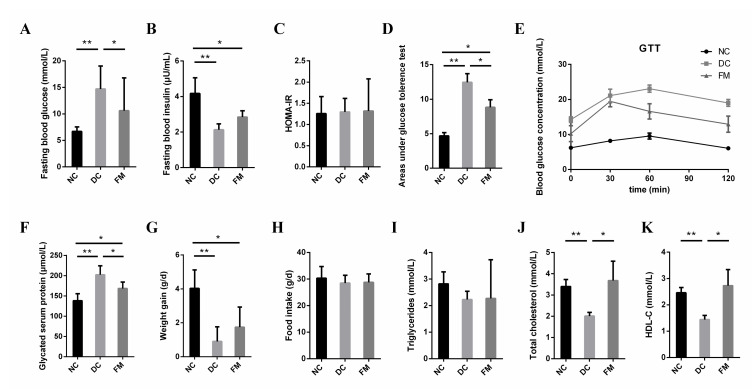
Effect of foxtail millet supplementation on glucose metabolism (**A**–**F**), weight gain (**G**), food intake (**H**), and lipid metabolism (**I**–**K**) in HFD/STZ-induced diabetic rats. Data were represented as mean ± SD. NC, normal control group (*n* = 8); DC, diabetic control group (*n* = 8); FM, foxtail millet supplementation group (*n* = 8); GTT, glucose tolerance tests; HDL-C, high-density lipoprotein cholesterol. Differences between groups were compared using one-way ANOVA with Tukey’s multiple comparison post hoc test, * *p* < 0.05, ** *p* < 0.01.

Compared with DC rats, the FBG concentration, GSP concentration, and areas under the GTTs (AUC) of FM rats were significantly decreased, while the TC and HDL-C concentration were significantly increased after 4 weeks of FM supplementation. There were no significant differences in the concentrations of the FBG, TC, and HDL-C between the NC and FM groups (Figure 1A,D,F,J,K). FM supplementation improved glucose tolerance significantly. Although the blood glucose concentration of FM rats was still higher than that of NC rats throughout GTT, the concentration decreased significantly at 0, 60, and 120 min when compared with DC rats (Figure 1E). However, FM supplementation did not cause significant improvements in the fasting blood insulin secretion, insulin resistance (HOMA-IR), body weight, food intake, and blood triglyceride concentration (Figure 1B,C,G–I).

### 3.2. FM Supplementation Changed the Gut Microbiota

To explore the role of the gut microbiota in the hypoglycemic effect of FM, we investigated the effects of FM supplementation on the composition and relative abundance of gut microbe using the 16S rRNA sequencing method. A total of 1,025,521 high-quality reads of 17 samples were generated, with an average of 56,973 ± 12,029 reads per sample. Based on 97% similarity, 787 OTUs were obtained, which could be divided into 12 phyla, 21 classes, 32 orders, 57 families, and 154 genera. The rarefaction and Shannon curves in Supplementary Materials Figure S1A,B indicated that the bacterial species were fully detected and evenly distributed. There were no significant differences in the richness (ACE) and α-diversity (Shannon and Simpson) of the gut microbiota among different groups (Supplementary Materials Figure S1C–E). 

**Figure 2:**
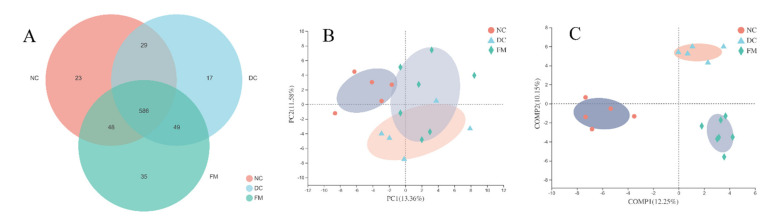
Effect of foxtail millet supplementation on the structure of gut microbiota: (**A**) Venn diagram on the OTU level; (**B**) unsupervised principal component analysis (PCA) on the OTU level; (**C**) supervised partial least squares discriminant analysis (PLS-DA) on the OTU level. NC, normal control group (*n* = 5); DC, diabetic control group (*n* = 5); FM, foxtail millet supplementation group (*n* = 7).

The Venn diagram illustrated that 586 out of 787 OTUs were shared among the three groups, while 35 OTUs were unique to the FM group (Figure 2A). To observe the effect of FM supplementation on the gut microbiota intuitively, we conducted both unsupervised principal component analysis (PCA) and supervised partial least squares discriminant analysis (PLS-DA). The PCA of three groups showed that the NC and DC groups were clearly clustered into two separate groups, while the FM group was clustered between them (Figure 2B). However, a completed separation of three groups in PLS-DA indicated significant differences among them (Figure 2C). These results suggested that FM supplementation significantly affected the gut microbial structure of diabetic rats. To a certain extent, this could alleviate the negative effects of diabetes on the gut microbiota.

**Figure 3:**
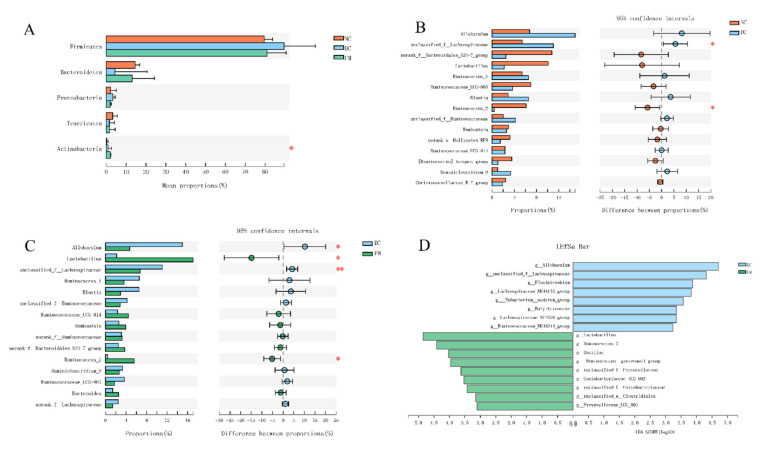
Effect of foxtail millet supplementation on the composition of gut microbiota: (**A**) phylum-level taxonomic distributions; (**B**,**C**) mean proportions of 15 key genera in different groups; (**D**) LDA scores derived from LefSe analysis, LDA > 3.0. Data were represented as mean ± SD. NC, normal control group (*n* = 5); DC, diabetic control group (*n* = 5); FM, foxtail millet supplementation group (*n* = 7). Differences between three groups were compared using one-way ANOVA with Tukey–Kramer post hoc test; differences between two groups were compared using Student’s *T*-test, * *p* < 0.05, ** *p* < 0.01.

Next, we performed a taxonomy-based analysis at the phylum and genus levels to evaluate the specific alterations of the gut microbe. The phylum *Firmicutes* was dominant among the 12 phyla presented in the gut microbiota from the three groups of mice with an average relative abundance of 83 ± 12% (Figure 3A). The ratio of *Firmicutes/Bacteroidetes* was significantly increased in DC rats, compared with NC rats, but not significantly decreased in FM rats (Figure S1F). The average compositions of bacterial communities with relative abundance higher than 1% at the genus level are shown in the Circos diagram (Figure S1G). 

There were significant differences in the abundance of *Lactobacillus*, *Ruminococcus_2* etc. among the three groups. Specifically, HFD, combined with STZ injection, significantly increased the relative abundances of *unclassified_f__Lachnospiraceae* and decreased the relative abundances of *Ruminococcus_2* in the DC group, as compared with the NC group (Figure 3B). By contrast, FM supplementation significantly decreased the relative abundances of *Allobaculum* and *unclassified_f__Lachnospiraceae* and increased the relative abundances of *Ruminococcus_2* and *Lactobacillus* in the FM group, as compared with the DC group (Figure 3C). There was no significant difference in the relative abundances of the above gut microbe between the NC and FM groups (Figure S1H). 

We then utilized the linear discriminant analysis (LDA) effect size (LEfSe) to identify further the specific bacterial taxa that significantly differed in response to FM supplementation. Compared with the DC rats, the FM rats had a higher abundance of *Lactobacillus* and *Ruminococcus_2* but a lower abundance of *Allobaculum* and *unclassified_f_Lachnospiraceae* (Figure 3D). 

### 3.3. FM Supplementation Reversed the Liver Transcriptomic Profiles 

To investigate the effect of FM supplementation on liver glucose metabolism, RNA-Seq was performed in the present study. The average alignment rate of the sequencing data was 95.48%, and more than 80% of the sequences were distributed in the coding region, indicating that the quality of the sequencing data met the requirements of the subsequent analysis. 

**Figure 4:**
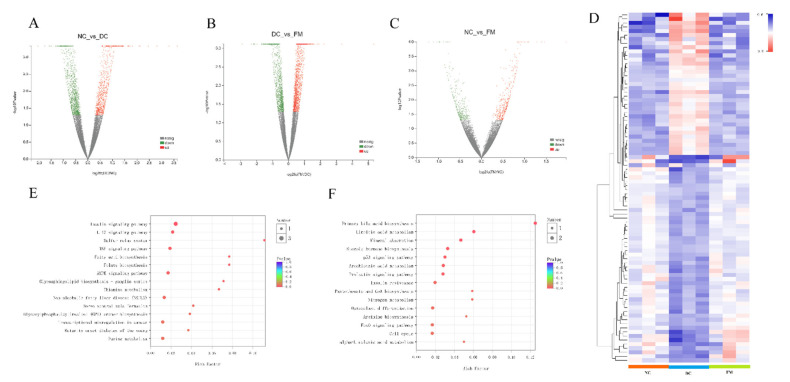
Effect of foxtail millet supplementation on liver transcriptomic profiles: (**A**–**C**) differentially expressed genes (DEGs) between different groups; (**D**) heatmap of 86 shared DEGs; (**E**) signaling pathways involved in upregulated DEGs; (**F**) signaling pathways involved in downregulated DEGs. NC, normal control group; DC, diabetic control group; FM, foxtail millet supplementation group.

The comparative analysis of the liver transcriptomic profiles indicated that there were 644 DEGs (Figure 4A–C). Among them, 230 DEGs were found between the DC and NC group (118 upregulated and 112 downregulated), and 487 DEGs were found between the FM and DC group (282 upregulated and 205 downregulated). There were only 32 DEGs found between the FM and NC rats (17 upregulated and 15 downregulated). We further screened and clustered the 86 shared DEGs between the NC-DC and DC-FM groups. As seen in the heatmap (Figure 4D), there were significant differences among the three groups. FM supplementation significantly reversed the trend of gene expression in diabetic rats. 

To investigate the signaling pathways involved in DEGs further, we used the KEGG database to perform the enrichment analysis. There were 103 signaling pathways involved in the shared DEGs. Compared with the DC group, the upregulated DEGs in the FM group were mainly involved in the insulin signaling pathway, IL-17 signaling pathway, and sulfur relay system (Figure 4E), while the downregulated DEGs were mainly involved in the primary bile acid biosynthesis, linoleic acid metabolism, and mineral absorption (Figure 4F). Among them, the insulin signaling pathway was the most affected by FM supplementation, which mainly referred to the insulin-induced PI3K/AKT signaling pathway in the KEGG pathway database.

### 3.4. FM Supplementation Activated the PI3K/AKT Signaling Pathway

**Figure 5:**
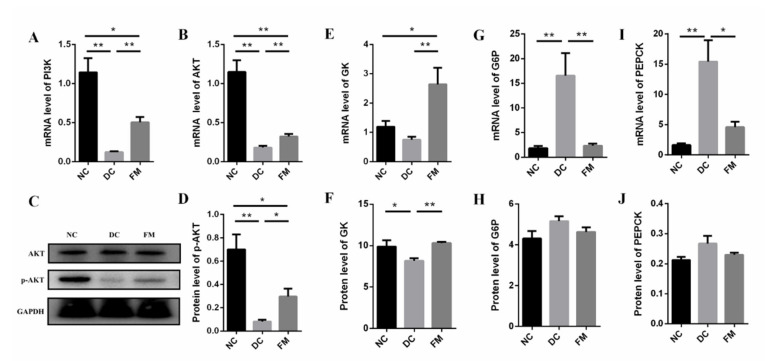
The expression of mRNA and protein of core factors in PI3K/AKT signaling pathway and its downstream effectors: (**A**–**D**) core factors in PI3K/AKT signaling pathway; (**E**,**F**) key enzymes in glycolysis; (**G**–**J**) key enzymes in gluconeogenesis. Data were represented as mean ± SEM. NC, normal control group; DC, diabetic control group; FM, foxtail millet supplementation group; PI3K, phosphatidylinositol-3-kinase; AKT, protein kinase B; G6P, glucose-6-phosphatase; GK, glucose kinase; PEPCK, phosphoenolpyruvate carboxy. Differences between groups were compared using Student’s *T*-test, * *p* < 0.05, ** *p* < 0.01.

The PI3K/AKT signaling pathway is the primary method for insulin to mediate glucose metabolism in the liver. Compared with the DC group, the expression of IRS (*Irs3*), PI3K (*Pik3r1*), and AKT (*Akt1*) were significantly upregulated after 4 weeks of FM supplementation (Table 1). Further analysis showed that the mRNA levels of PI3K and AKT in the liver tissue of DC rats were significantly lower than those of NC and FM rats. At the protein level, there was no significant difference in the AKT expression among the three groups. However, the expression of phosphorylated AKT (p-AKT—activated AKT) in the FM group was significantly higher, compared with the DC group (Figure 5A–D). These results indicate that FM supplementation activated the PI3K/AKT signaling pathway.

Glycolysis and gluconeogenesis are the key steps to maintain blood glucose homeostasis in liver tissue. The RNA-Seq results showed that the key enzymes in glycolysis, GK, and pyruvate kinase (PK) in the FM group were significantly higher, compared with that of the DC group, which was consistent with the validation results of GK at both gene and protein levels (Table 1, Figure 4E,F). Compared with the DC group, the key enzymes in gluconeogenesis, G6P, and PEPCK were significantly decreased at the gene level (Figure 4G–I). 

In addition, insulin also regulated the lipid metabolism in liver tissue. In the present study, the expression levels of the lipid-synthesis-related genes of the DC group were significantly lower than those of the NC group, such as sterol regulatory element-binding protein-1c (SREBP1c), acetyl-CoA carboxylase (ACC), and fatty acid synthase (FAS). However, these lipid-synthesis-related genes were significantly upregulated after 4 weeks of FM supplementation (Table 1); that is to say, FM supplementation could inhibit gluconeogenesis, stimulate glycolysis, and restore fatty acid synthesis through activating the PI3K/AKT signaling pathway.

### 3.5. FM Supplementation Reduced Inflammation by Inhibiting NF-κB Signaling Pathway

**Figure 6:**
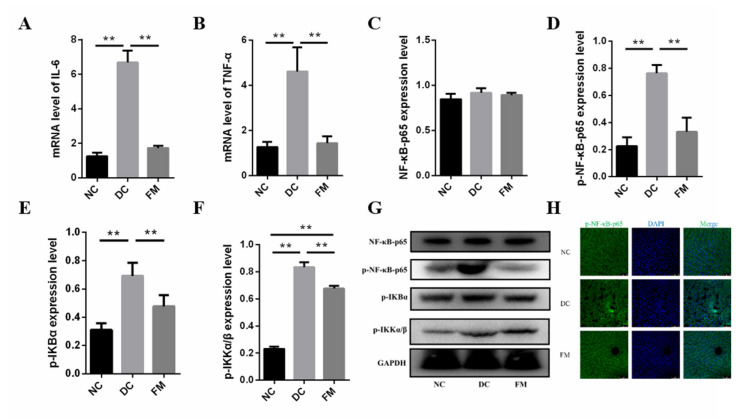
The expression of inflammatory cytokines (**A**,**B**), core factors in NF-κB signaling pathway (**C**–**G**), and nuclear translocation of p- NF-κB-p65 (**H**). Data were represented as mean ± SEM. NC, normal control group; DC, diabetic control group; FM, foxtail millet supplementation group; IΚB, κB kinase; IKK, inhibitor of κB kinase. Differences between groups were compared using Student’s *T*-test, * *p* < 0.05, ** *p* < 0.01.

T2D is associated with low-grade inflammation [19], which was confirmed again in our present study. The concentrations of IL-6 and TNF-α in the DC group were significantly higher than those in the NC group. After 4 weeks of FM supplementation, the concentrations of IL-6 and TNF-α were significantly decreased (Figure 6A,B). 

The NF-κB signaling pathway is the primary method of the inflammatory response [20]. Thus, we further investigated the protein expression involved in the NF-κB signal pathway. Although there was no significant difference of the total NF-κB-p65 in the cytoplasm among the three groups, the expression of p-NF-κB-p65, the phosphorylated inhibitor of κB kinase (p-IKKα/β), and the phosphorylated κB kinase (p-IΚBα) in the DC group were significantly higher than those in the NC group. The expression of these phosphorylated proteins was significantly decreased again after the FM supplementation (Figure 6C–G). In addition, the fluorescence of p-NF-κB-p65 in both the cytoplasm and nucleus of the DC group was significantly enhanced. However, no significant nuclear translocation was found in the FM and NC groups (Figure 6H). These results suggest that FM supplementation could reduce inflammation by inhibiting the NF-κB signaling pathway.

### 3.6. Correlations among the Bacteria, Signaling Pathways, and Metabolic Parameters

**Figure 7:**
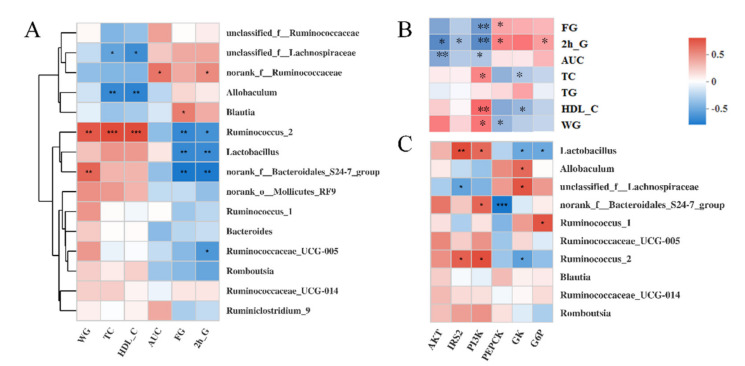
Heatmap of Spearman’s correlation analysis between gut microbiota (top 15 at genus level) and metabolic parameters (**A**), liver gene expression and metabolic parameters (**B**), gut microbiota (top 10 at genus level), and liver gene expression (**C**). Significant correlations are marked by * *p* < 0.05; ** *p* < 0.01. IRS, insulin receptor substrate; PI3K, phosphatidylinositol-3-kinase; AKT, protein kinase B; GK, glucose kinase; G6P, glucose-6-phosphatase; PEPCK, phosphoenolpyruvate carboxy; WG, weight gain; TC, total triglycerides; FG, fasting glucose; HDL-C, high-density lipoprotein cholesterol; AUC, areas under the glucose tolerance test.

Spearman’s correlation analysis was performed to explore further the correlation between the gut microbiota (the top 15 at the genus level) and metabolic parameters (Figure 7A). The results showed that the FM-supplementation-enriched *Ruminococcus_2* and *Lactobacillus* were significantly negatively correlated with the FG and 2-h G. The DC rats enriched *Allobaculum* and *unclassified_f__Lachnospiraceae* were significantly negatively correlated with the TC and HDL-C. Moreover, *Ruminococcus_2* also had a significant positive correlation with the WG, TC, and HDL-C. 

We also performed Spearman’s correlation analyses between six key genes and seven physiological indices (Figure 7B). The results showed that these seven physiological indices were divided into two subgroups with opposing correlation results. Specifically, there was a significant negative correlation between the expression of PI3K and the concentration of FG, 2-h G, and the AUC. Similar correlation results were observed between AKT and the 2-h G, AKT, AUC, IRS2, and 2h-G. The expression of PI3K was significantly positively associated with the concentration of TC, HDL-C, and WG. Moreover, the expression of PEPCK was significantly positively associated with the concentration of FG and 2-h G but negatively associated with the concentration of WG.

To analyze further the association between the gut microbiota and the liver gene expression, we then performed correlation analyses between 6 key genes and the top 10 gut microbes at the genus level (Figure 7C). Similar to *Ruminococcus_2*, the relative abundance of *Lactobacillus* was significantly positively associated with the expression of PI3K and IRS2 and negatively associated with GK and G6P. Conversely, there was a significant negative correlation between the IRS2 expression and the *unclassified_f__Lachnospiraceae* abundance, which was positively correlated with the GK expression.

## 4. Discussion

Over the past few decades, numerous studies have demonstrated unambiguous evidence regarding the role of whole-grain consumption in improving blood glucose metabolism and preventing T2D [3]. However, the research on the health benefit effects of whole grains is far from sufficient particularly regarding the molecular mechanisms [4]. FM is one of the most important drought-resistant crops. It holds an immense assurance for food safety and nourishment in the arid and semiarid areas of Asia and Africa [5]. 

Our previous studies demonstrated that FM had a relatively low starch digestibility and moderate glycemic indices [7]. Habitual FM consumption could improve glycemic control in subjects with impaired glucose tolerance [8]. In the present study, the hypoglycemic effect of FM supplementation was confirmed again in HFD/STZ diabetic rats by the significantly decreased FG, GSP, and AUC. Tremendous attention has been given to understanding the underlying mechanism.

Dysfunctional gut microbiota have been considered one of the causes for a series of metabolic disorders, including T2D. Although the exact mechanism linking the gut microbiota to glucose homeostasis is far from being well understood, a substantial body of research has provided evidence for the important role of the gut microbiota in glucose metabolism [9,10]. The results in the present study showed that FM supplementation partially reversed the adverse changes of the gut microbiota in diabetic rats. Specifically, FM supplementation significantly increased the relative abundance of *Lactobacillus* and *Ruminococcus_2* and significantly decreased the relative abundance of *Allobaculum* and *unclassified_f_Lachnospiraceae*. 

*Lactobacillus* is the most used probiotic in research and clinical settings. The beneficial effects on the gastrointestinal tract and immune system as well as the metabolic properties have been widely reported [23]. A substantial body of literature has provided evidence for the positive role of Lactobacillus in T2D. For example, *Lactobacillus casei* CCFM419 was shown to favorably regulate the blood glucose balance, increase glucose tolerance, and protect islets in diabetic mice through the underlying PI3K/AKT signaling pathway [20]. *Lactobacillus plantarum* Ln4 significantly stimulated glucose uptake in 3T3-L1 adipocytes, attenuated insulin resistance, and changed the hepatic mRNA levels (IRS and AKT) associated with glucose metabolism [24]. In addition, the hypoglycemic benefits of *Lactobacillus* were, at least in part, via changes in the microbiota composition and intestinal barrier. *Lactobacillus plantarum* X1 improved glucose tolerance by increasing the abundance of butyric-acid-producing bacteria [25]. *Lactobacillus reuteri* GMNL-263 supplementation decreased the pathogen abundances and improved the intestinal barrier [26]. 

Although *Ruminococcus* was reported in a positive association with T2D [9], it plays an important role in the biodegradation of resistant starch and other dietary fiber [27]. The FM used in this study was a whole grain food with a high content of resistant starch [7], which thus increased the relative abundance of *Ruminococcus*. *Allobaculum* demonstrated a high abundance in the gut of mice fed a high-fat diet [28], which was in accordance with our result of DC rats. A previous study reported that *unclassified_f_Lachnospiraceae* could significantly increase FG and reduce insulin sensitivity [29]. Interestingly, FM supplementation could reverse these adverse increases in diabetic rats. Collectively, the above results suggest that the hypoglycemic effect of FM was at least partially mediated by structural modulation of the gut microbiota.

The liver plays an important role in maintaining blood glucose homeostasis [30]. Liver glucose metabolism includes glucose transport, glycolysis, gluconeogenesis, hepatic glycogen synthesis, and decomposition [31], which were regulated by the insulin-mediated PI3K/AKT signaling pathway [15]. Therefore, to understand the underlying mechanism of the beneficial role of FM on glucose metabolism, we investigated the specific effect of FM supplementation on the PI3K/AKT signaling pathway and its downstream effectors. 

Based on the accumulated evidence, the PI3K/AKT signaling pathway is the major effector of insulin in regulating metabolism [15]. Damage to the PI3K/AKT signaling pathway in liver tissues would thus lead to insulin resistance and, thereby, T2D. In turn, insulin resistance would exacerbate the PI3K/AKT signaling pathway, forming a vicious cycle [30]. IRS, PI3K, and AKT are the most critical factors in the PI3K/AKT signaling pathway. For example, AKT1 is ubiquitously expressed, with high levels in classical insulin target tissues, such as the liver. Studies have shown the positive role of AKT1 in the improvement of insulin sensitivity and decrease of blood glucose [32]. In the present study, the activation of the PI3K/AKT signaling pathway of FM supplementation was suggested by the significantly upregulated expression of IRS, PI3K, and AKT. 

However, both the core factors in the PI3K/AKT signaling pathway and their downstream effectors perform distinct functions in the regulation of glucose homeostasis [15]. We then analyzed the expression profiles of the downstream effectors to clarify further the biological process and target genes of FM related to the hypoglycemic effect. Glycolysis and gluconeogenesis are two key steps involved in maintaining blood glucose homeostasis, which involves many key catalytic enzymes, such as G6P in glycolysis and GK in gluconeogenesis. 

Insulin and phytochemicals could improve glucose metabolism by regulating the activity and expression of these enzymes [33,34]. For example, polysaccharide fromDendrobium officinale was shown to reduce the blood glucose concentration via the regulation of glucose metabolizing enzyme activity, including PK, hexokinase (HK), and PEPCK in the liver [34]. Our results showed that the expressions of GK and PK in the liver tissue of the FM group were 1.53-fold and 2.27-fold higher than those of the DC group, indicating that FM supplementation promoted glycolysis in the liver tissue of diabetic rats by upregulating the expression of the key enzymes GK and PK. 

FoxO1 is the main inhibition target of AKT. FoxO1 induces the expression of PEPCK and G6P and subsequently increases gluconeogenesis in liver tissue [30]. A previous study showed that fructose administration enhanced the phosphorylation of FoxO1 and then suppressed the gluconeogenic gene expression, G6P activity, and glucose production from pyruvate [18]. Our results also indicated that FM supplementation suppressed gluconeogenesis by downregulating the expression of G6P, FBP, and PEPCK and then inhibiting the production and release of glucose from the liver. 

As with the regulation of glucose metabolism, insulin can also stimulate fatty acid synthesis and inhibit the decomposition in normal hepatocytes via the PI3K/AKT signaling pathway [33]. However, in insulin-resistant hepatocytes, the regulation of the insulin signaling pathway is damaged [35]. SREBP1c is a major regulator of fatty acid synthesis. AKT could promote the production of FAS and ACC by upregulating the expression of SREBP1c [30,36]. Previous studies demonstrated the downregulation of the expression of SREBP1c in STZ-induced diabetic rats [37], which was consistent with our results. In the present study, 4 weeks of FM supplementation significantly upregulated the expression of SREBP1c, FAS, and ACC. FM supplementation repaired the impaired fatty acid synthesis function of diabetic rats and improved the balance of the energy metabolism.

In addition, inflammation is a common feature of T2D [19]. This was proven again by the increased inflammatory cytokines of the DC rats in the present study. However, FM supplementation significantly reduced the inflammation of diabetic rats. Considering the important role of the NF-κB signaling pathway in the inflammatory response [38], we detected the expression and translocation of key effectors in the NF-κB signaling pathway. The results showed that FM supplementation significantly reduced the phosphorylation levels of IKK, IκB, and NF-κB in the liver cells of diabetic rats. Immunofluorescence showed that there was no significant difference in the level of p-NF-κB-p65 between the NC group and FM group; however, the levels were significantly lower than for the DC group. The phosphorylation of IKK, IκB, and NF-κB and the entry of p-NF-κB-p65 from the cytoplasm into the nucleus are necessary for the activation of the NF-κB signaling pathway [38]. Therefore, FM supplementation could significantly reduce inflammation by suppressing the activation of the NF-κB signaling pathway. 

Previous studies demonstrated that the activation of NF-κB was related to blood glucose control in diabetic patients. Inhibition of the NF-κB signaling pathway and the reduction of inflammation have been proven to be beneficial to blood glucose homeostasis [39]. Specifically, IKK decreased insulin sensitivity by catalyzing the serine phosphorylation of IRS and inhibiting its tyrosine phosphorylation [40]. The inhibition of IKK activity was shown to promote AKT phosphorylation and thereby improve blood glucose metabolism [41]. Inflammatory cytokines, such as TNF-α and IL-6, can also inhibit insulin signal transduction in hepatocytes [42].

**Figure 8:**
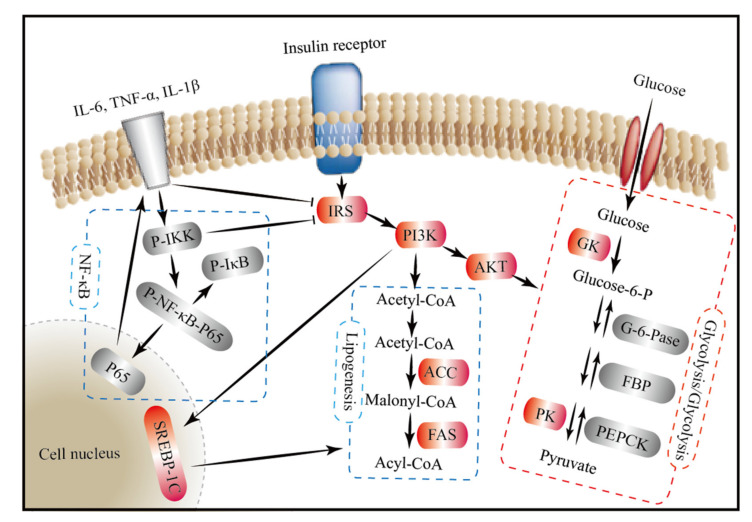
The mechanisms underlying the hypoglycemic effect of foxtail millet from the perspective of signaling pathways. Foxtail millet supplementation might improve the blood glucose metabolism by inhibiting gluconeogenesis, stimulating glycolysis, and repairing fatty acid synthesis through the insulin-mediated PI3K/AKT signaling pathway, as well as reducing inflammation through the NF-κB signaling pathway. Red background, significantly upregulated genes or proteins; grey background, significantly downregulated genes or proteins; arrow for promotion and —| for inhibition; IRS, insulin receptor substrate; PI3K, phosphatidylinositol-3-kinase; AKT, protein kinase B; GK, glucose kinase; PK, pyruvate kinase; G6P, glucose-6-phosphatase; FBP, fructose bisphosphatase; PEPCK, phosphoenolpyruvate carboxy; FAS, fatty acid synthase; ACC, acetyl-CoA carboxylase; SREBP1c, sterol regulatory element-binding protein-1c; IΚB, κB kinase; IKK, inhibitor of κB kinase.

In summary, we propose a molecular mechanism of the hypoglycemic effect of FM from the perspective of signaling pathways (Figure 8). On the one hand, FM supplementation might activate the PI3K/AKT signaling pathway by upregulating the expression of IRS, PI3K, and AKT and thereby promote glycolysis by upregulating the expression of GK and PK; inhibit gluconeogenesis by downregulating the expression of G6P, FBP, and PEPCK; and repair impaired fatty acid synthesis by upregulating the expression of FAS and ACC. On the other hand, FM supplementation might improve the blood glucose metabolism by inhibiting the NF-κB signaling pathway and reducing the expression of inflammatory cytokines, which can stimulate the activation of the insulin signaling pathway.

Human genes, microbial genes, and the diet share a complicated set of interdependencies [43]. Although dissecting the role of the gut–liver axis in glucose metabolism is a great challenge, the link between these is becoming clearer with increasing studies showing the involvement of the gut microbiota in insulin signaling and low-grade inflammation [10]. For example, *Lactobacillus casei* activated the PI3K/AKT signaling pathway by increasing the mRNA level of PI3K, IRS, and AKT in liver tissue [20]. *Lactobacillus paracasei* inhibited the NF-κB signaling pathway by suppressing the expression of IL-6 and TNF-α [9]. The relative abundance of *Lactobacillus* was also significantly positively associated with the expression of PI3K and IRS in the present study.

This study is a preliminary study on the hypoglycemic mechanism of foxtail millet (FM). Both the gut microbiota and the liver gene transcriptome are very complex systems. The FM is also a multicomponent complex. Although our study established a link between the hypoglycemic effect of FM, the relative abundance of Lactobacillus and PI3K/AKT signaling pathway, the specific association mechanisms among FM supplementation, glucose metabolism, the gut microbiota, and the signaling pathway still need to be further clarified. For example, what are the key components of FM for hypoglycemic effect, and how do these key components affect the PI3K/AKT signaling pathway?

## 5. Conclusions

Based on the confirmatory hypoglycemic effect of FM supplementation, tremendous attention has been given to understanding the underlying molecular mechanisms. Collectively, FM supplementation might improve the blood glucose metabolism by (a) modulating the structure of the gut microbiota, particularly by increasing the relative abundance of *Lactobacillus*; (b) inhibiting gluconeogenesis, stimulating glycolysis, and repairing fatty acid synthesis through the insulin-mediated PI3K/AKT signaling pathway; and (c) reducing inflammation through the NF-κB signaling pathway. However, the internal relationship among these different mechanisms requires further study.

## Figures and Tables

**Table 1:** Expression levels of key genes in PI3K/AKT signaling pathway.

Name	Gene	Expression (FPKM)			Expression Fold		
		NC	DC	FM	DC/NC	FM/DC	FM/NC
PI3K/AKT signaling pathway							
Insulin receptor substrate (IRS)	*Irs3*	3.11	1.48	3.95	0.57 *	1.85 *	1.11
Phosphatidylinositol-3-kinase (PI3K)	*Pik3r1*	18.02	11.66	23.07	0.71 *	1.79 *	1.23
Protein kinase B (AKT)	*Akt1*	28.54	25.69	31.06	0.85	1.26 *	1.07
Glycolysis/Gluconeogenesis							
Glucose kinase (GK)	*Gck*	25.66	31.62	50.95	1.10	1.53 *	1.47 *
Pyruvate kinase (PK)	*Pklr*	54.18	47.68	113.96	0.90	2.27 *	1.54 *
Fructose bisphosphatase (FBP)	*Fbp1*	582.91	703.23	525.34	1.12	0.80 *	0.90
	*Fbp2*	0.45	0.77	0.22	1.26	0.29 *	0.83
Lipid synthesis							
Sterol regulatory element-binding protein-1c (SREBP1c)	*Srebf1*	58.18	36.01	138.23	0.67 *	2.66 *	1.71 *
Acetyl-CoA carboxylase (ACC)	*Acaca*	6.29	2.67	10.98	0.50 *	2.89 *	1.34
Fatty acid synthase (FAS)	*Fasn*	4.51	1.14	13.77	0.40 *	2.45 *	1.24

Note: NC, normal control group; DC, diabetic control group; FM, foxtail millet supplementation group. * *p* < 0.05.

## Data Availability

All data that support the findings of this study are available from the corresponding author on reasonable request.

## Supplementary Materials

The following are available online at https://www.mdpi.com/article/10.3390/nu13061837/s1, Table S1: Composition and energy ratio of experimental diets, Table S2: Primers for PCR, Figure S1: Changes in the structure and composition of gut microbiota.
