# Supplementary material for: Foxtail Millet Improves Blood Glucose Metabolism in Diabetic Rats through PI3K/AKT and NF-κB Signaling Pathways Mediated by Gut Microbiota
Source: Nutrients. 2021 May 27;13(6):1837. doi: 10.3390/nu13061837 (PMC8228963; doi:10.3390/nu13061837)
Supplement: Supplementary file 1 [file nutrients-13-01837-s001.zip › nutrients-1210266-supplementary.pdf]

Table S1. Composition and energy ratio of experimental diets.

Ingredient (g/100g)	control diet	High-fat diet	20% foxtail millet diet
Foxtail Millet	-	-	20.00
Casein	18.96	25.85	23.85
L-Cystine	0.28	0.39	0.39
Corn Starch	47.98	-	-
Maltodextrin	11.85	16.15	8.15
Sucrose	6.52	8.89	3.89
Cellulose	4.74	6.46	2.46
Soybean Oil	2.37	3.23	3.23
Lard	1.90	31.66	30.66
Mineral Mix M1002	0.95	1.29	1.29
DiCalcium Phosphate	1.23	1.68	1.68
Calcium Carbonate	0.52	0.71	0.71
Potassium Citrate	1.56	2.13	2.13
Vitamin Mix V1001	0.95	1.29	1.29
Choline Bitartrate	0.19	0.26	0.26
**Energy Ratio**			
% Energy from protein	20	20	20
% Energy from fat	10	60	60
% Energy from carbohydrate	70	20	20

To guarantee consistent energy ratios among 20% FM and high-fat diets, the content of casein, maltodextrin, sucrose, cellulose, and lard were reduced accordingly in the 20% FM diet.

Table S2. Primers for PCR.

Name	Sequence (5′-3′)	Length
IL-6-F	GAAATACAAAGAAATGATGG	20
IL-6-R	GTGTTTCAACATTCATATTGC	21
TNF-α-F	TACTGAACTTCGGGGTGATTGGTCC	25
TNF-α-R	CAGCCTTGTCCCTTGAAGAGAACC	24
IRS2-F	CCACCTTCTCTGGCAGTTCAG	21
IRS2-R	AAGGGTTGTAGGCCACTTTGG	21
PI3K(p110)-F	GGAGGCTGAATCTCTTGACCTG	22
PI3K(p110)-R	TTACCCACCGTGCTCTGCTG	20
AKT-F	GTTCTCCTACTCAGCCAGTG	20
AKT-R	CCATCAGTGACAGATGATCC	20
GK-F	GTGGTGCTTTTGAGACCCGTT	21
GK-R	TTCGATGAAGGTGTATTCGCA	21
G6P-F	TTTATCCTACCATCAGGTGGCTGT	24
G6P-R	GGGTGACAGATACTTCGCTAGTTG	24
PEPCK-F	ACTGAGCAGCCCTTGTGACA	20
PEPCK-R	GAGGGCGTGAAGTAC	18

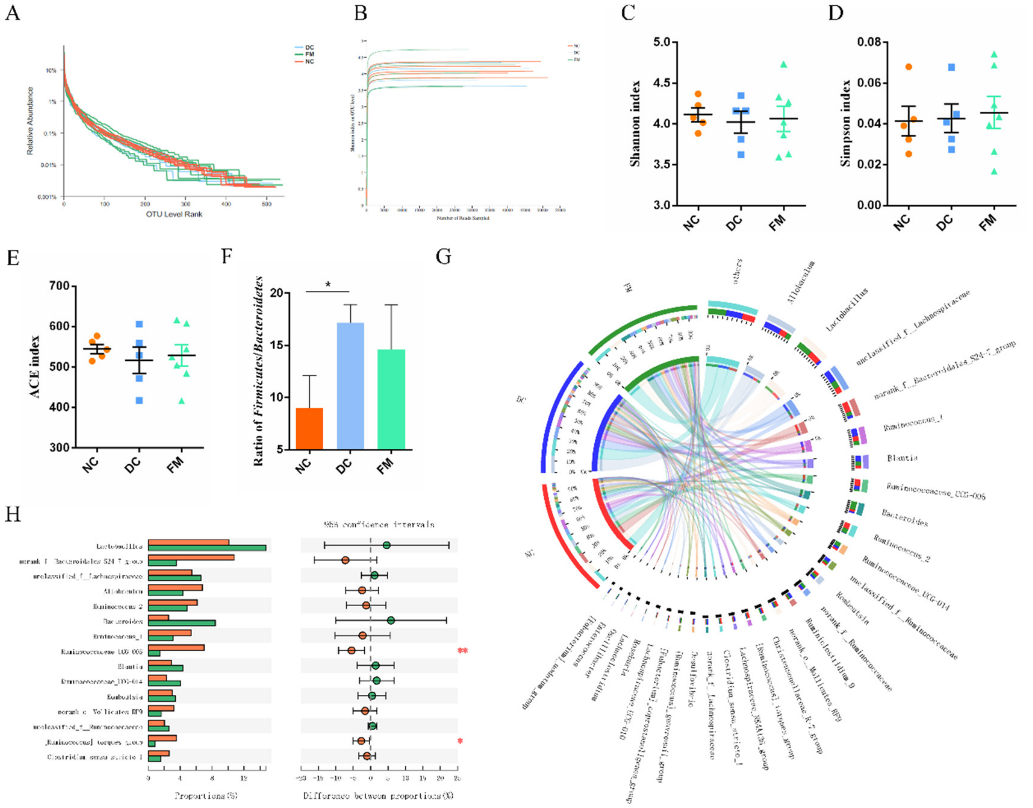

Figure S1. Changes in the structure and composition of gut microbiota: (A) the rarefaction curve; (B) the Shannon curve; (C–E) the richness (ACE) and α-diversity (Shannon and Simpson) of the gut microbiota among different groups; (F) the ratio of *Firmicutes*/*Bacteroidetes*; (G) Circos diagram of bacterial communities with relative abundance higher than 1% at the genus level; (H) mean proportions of top 15 key genera between NC and FM groups. Data were represented as mean ± SEM. NC, normal control group (n=5); DC, diabetic control group (n=5); FM, foxtail millet supplementation group (n=7). Differences between two groups were compared using Student's *t*-test, *$p < 0.05$, **$p < 0.01$.
